# What killed Karl Patterson Schmidt? Combined venom gland transcriptomic, venomic and antivenomic analysis of the South African green tree snake (the boomslang), *Dispholidus typus*

**DOI:** 10.1016/j.bbagen.2017.01.020

**Published:** 2017-04

**Authors:** Davinia Pla, Libia Sanz, Gareth Whiteley, Simon C. Wagstaff, Robert A. Harrison, Nicholas R. Casewell, Juan J. Calvete

**Affiliations:** a Laboratorio de Venómica Estructural y Funcional, Instituto de Biomedicina de Valencia, CSIC, Valencia, Spain; b Alistair Reid Venom Research Unit, Parasitology Department, Liverpool School of Tropical Medicine, Liverpool, United Kingdom; c Bioinformatics Unit, Parasitology Department, Liverpool School of Tropical Medicine, Liverpool, United Kingdom

**Keywords:** Boomslang, *Dispholidus typus*, Non-front-fanged colubroid snake venomics, Snake venom gland transcriptomics, Antivenomics, Disseminated intravascular coagulation/venom induced consumption coagulopathy

## Abstract

**Background:**

Non-front-fanged colubroid snakes comprise about two-thirds of extant ophidian species. The medical significance of the majority of these snakes is unknown, but at least five species have caused life-threatening or fatal human envenomings. However, the venoms of only a small number of species have been explored.

**Methods:**

A combined venomic and venom gland transcriptomic approach was employed to characterise of venom of *Dispholidus typus* (boomslang), the snake that caused the tragic death of Professor Karl Patterson Schmidt. The ability of CroFab™ antivenom to immunocapture boomslang venom proteins was investigated using antivenomics.

**Results:**

Transcriptomic-assisted proteomic analysis identified venom proteins belonging to seven protein families: three-finger toxin (3FTx); phospholipase A_2_ (PLA_2_); cysteine-rich secretory proteins (CRISP); snake venom (SV) serine proteinase (SP); C-type lectin-like (CTL); SV metalloproteinases (SVMPs); and disintegrin-like/cysteine-rich (DC) proteolytic fragments. CroFab™ antivenom efficiently immunodepleted some boomslang SVMPs.

**Conclusions:**

The present work is the first to address the overall proteomic profile of *D. typus* venom. This study allowed us to correlate the toxin composition with the toxic activities of the venom. The antivenomic analysis suggested that the antivenom available at the time of the unfortunate accident could have exhibited at least some immunoreactivity against the boomslang SVMPs responsible for the disseminated intravascular coagulation syndrome that caused K.P. Schmidt's fatal outcome.

**General significance:**

This study may stimulate further research on other non-front-fanged colubroid snake venoms capable of causing life-threatening envenomings to humans, which in turn should contribute to prevent fatal human accidents, such as that unfortunately suffered by K.P. Schmidt.

## Highlights

• The venom proteome of *Dispholidus typus* (boomslang) is reported.
• Transcriptomic-assisted proteomic analysis identified venom proteins belonging to seven protein families.
• Boomslang venom proteome is dominated (75%) by snake venom PIII-metalloproteinases (PIII-SVMPs).
• CroFab™ antivenom efficiently immunodepleted some boomslang PIII-SVMPs.

## 1 Introduction

Karl Patterson Schmidt (June 19, 1890, Lake Forest, Illinois–September 26, 1957, Chicago) was an eminent American herpetologist at the Field Museum in Chicago, where he was zoological curator from 1941 to 1955, and a leading expert on coral snakes. He died nearly 6 decades ago of internal bleeding from his eyes, lungs, kidneys, heart, and brain 24 h after being bitten on his thumb by a juvenile South African green tree snake, also known as the boomslang [1]. The snake had been sent to him for identification by the then director of the Lincoln Park Zoo (Chicago. IL), Richard Marlin Perkins [2].

Despite studies by Grasset et al. (1940) [3] having shown marked procoagulant activity associated with *D. typus* envenomation *in vivo*, and serious human envenomations by boomslangs having been documented [4], [5], [6], K. P. Schmidt, along with many of his contemporary herpetologists, wrongly believed that rear-fanged colubroid snakes could not produce a fatal venom dose for humans. With this conviction, and guided by his scientific curiosity, following the bite he made meticulous notes about the effects he experienced as the venom took hold of his body. Schmidt was asked just a few hours before he died if he wanted medical care, but he refused because it would upset the symptoms he was documenting. Science Friday (http://www.sciencefriday.com) has released the video “Diary of a snakebite death‬” (https://www.youtube.com/watch?v=jEyjF2bNQOA) detailing, in his own words and using his notes, the last hours of Schmidt's life. We have here used the published facts of this tragic event to emphasise the important contributions that modern biological science can make to clinical medicine, and have not used any personal material or data in our analyses.‬‬‬‬‬‬‬‬‬‬‬‬‬‬‬‬‬‬‬‬‬‬‬‬‬‬

The boomslang, *Dispholidus typus* (Smith, 1828) [7], “tree snake” in Afrikaans and Dutch, is a sub-Saharan African indigenous species widely distributed throughout much of the central and southern regions of the continent, including Swaziland, Botswana, Namibia, Mozambique, and Zimbabwe, but has been observed as far north as Nigeria and southern Chad, and as far east as Kenya [8], [9]; http://reptile-database.reptarium.cz]. Boomslangs are a large (average adult total length is 100–160 cm, but some exceed 183 cm), diurnal and almost exclusively arboreal opisthoglyphous (rear-fanged) colubroid snake species (Colubridae: Colubrinae). Boomslangs feed on chameleons and other arboreal lizards, frogs, and occasionally small mammals, birds, and eggs from nesting birds [8], [9], [10]. The boomslang is a mellow, shy snake that will flee from anything too large to eat, and will bite only when people attempt to handle, catch or kill the animal [8], [9], [10].

Today, the boomslang is considered one of the most deadly African snakes, and is probably responsible for more serious bites in humans than any other non-front-fanged colubroid species. Boomslangs have very long fangs and can open their mouths a full 180 degrees to bite. An adult boomslang has 1.6–8 mg of venom, whose median lethal dose (LD_50_) in mice is 0.07–0.1 mg/kg (i.v.), 12.5 mg/kg (s.c.), and 1.3–1.8 mg/kg (i.p.) [11], [12], [13]. Typical symptoms associated with envenomings by *D. typus* include disseminated intravascular coagulation (DIC)-like syndrome (also referred to as venom induced consumption coagulopathy) with delayed onset of spontaneous haemorrhage into tissues [9], [10], [14], [15], [16], [17]. Renal failure may also result from acute tubular necrosis caused by pigment nephropathy. DIC is caused by the consumption of fibrinogen (defibrination) into many small clots, resulting in blood that loses the ability to clot and thus the victim bleeds to death. An indication of the important health issues associated with *D. typus* is that its venom is the only colubroid snake against which the South African Vaccine Producers manufactures a commercial monovalent antivenom [18].

Despite comprising approximately two-thirds of the described species of advanced snakes, venoms of non-front-fanged colubroid snakes have not been extensively investigated. Modest information on the composition of boomslang venom is available. Gel-based assays demonstrated the presence of phospholipase A_2_ (PLA_2_) [19], [20], caseinolytic [21] and gelatinase [7] activities. A 65 kDa snake venom metalloproteinase (SVMP) bearing antigenic determinants recognised by Western blot by polyclonal antibodies raised against the haemorrhagic PIII-SVMP jararhagin (isolated from the venom of the Brazilian pitviper *B. jararaca*) was also demonstrated to be present in boomslang venom [22]. Proteins consistent with the masses and retention times of three-finger toxins (3FTxs) (7–8 kDa), PLA_2_ (13–14 kDa) and cysteine-rich secretory proteins (CRISP) (25 kDa) have been identified in a number of non-front-fanged colubroid venoms, including *D. typus*, by liquid chromatography and mass spectrometry [23]. However, venoms act as integrated phenotypes, and a detailed view of the venom proteome of this medically important snake remains lacking. Here, we have applied a combined snake venomics and venom gland transcriptomic approach [24] to fill this knowledge gap.

## 2 Materials and methods

### 2.1 Venom and venom gland

Lyophilised venom from specimens of *D. typus* of South African origin was obtained from Latoxan, France and stored at 4 °C until use. The venom gland was dissected from a single euthanised specimen of *D. typus* of Tanzanian origin that was maintained in the herpetarium of the Liverpool School of Tropical Medicine, UK. Dissected venom glands were immediately flash frozen in liquid nitrogen and stored cryogenically until use.

### 2.2 Characterisation of the venom gland transcriptome

We generated a venom gland transcriptome in a similar manner to those previously described by our group [25], [26]. The venom gland was first homogenised under liquid nitrogen using a pestle and mortar and then subsequently by using a TissueRuptor (Qiagen), before total RNA was extracted using the TRIzol Plus RNA Purification kit (Life Technologies, Carlsbad, CA, USA). The RNA sample was then DNAse treated (On-Column PureLink DNase, Life Technologies) and 1 μg of the resulting RNA enriched using a polyA selection method (Dynabeads mRNA Purification Kit from total RNA preps, ThermoFisher Scientific). The RNA-Seq library was prepared from 50 ng of the enriched RNA material using the TruSeq Stranded mRNA HT Sample Prep Kit (Illumina). During the preparation, dual index adapters were used so that other RNA samples in the sequencing pool (not described here) had a unique index at both the 5′ and 3′ ends. The sequencing library was amplified with 12 cycles PCR and then purified using AMPure XP beads (Agencourt, Brea, CA, USA), quantified using the Qubit dsDNA HS Assay Kit (Life Technologies) and the size distribution assessed using a Bioanalyser (Agilent). The quantity and quality of the resulting sample was also assessed by qPCR using the Illumina Library Quantification Kit on a Roche Light Cycler LC480II according to manufacturer's instructions. Finally, the template DNA was denatured according to the protocol described in the Illumina cBot User guide and loaded at 10 pM concentration. Sequencing was carried out on a single lane of an Illumina MiSeq with 2 × 250 bp paired-end sequencing and v2 chemistry (Centre for Genomic Research, University of Liverpool, UK).

The ensuing read data was quality processed, first by removing the presence of any adapter sequences using Cutadapt (https://code.google.com/p/cutadapt/) and then by trimming low quality bases using Sickle (https://github.com/najoshi/sickle). Reads were trimmed if bases at the 3′ end matched the adapter sequence for 3 bp or more, and further trimmed with a minimum window quality score of 20. After trimming, reads shorter than 10 bp were removed. Processed paired-end read data were next assembled into contigs using the *de novo* transcriptome assembler VTBuilder [27] executed with the following parameters: min. transcript length 150 bp; min. read length 150 bp; min. isoform similarity 96%. The isoform similarity parameter is a standard parameter optimised to discriminate between isoforms excepting minor polymorphisms in mixed specimen samples and sequencing errors. Raising this to close to 100% could potentially return a small number of transcripts between 96 and 100% identity that have been merged during the assembly process but can be detrimental to the quality (both length and representation) of the final assembly due to the effects of under-clustering driven by sequencing or other minor polymorphic variations.

Assembled contigs were annotated with BLAST2GO Pro v3 [28], [29] using the BLASTX algorithm with a significance threshold of 1e − 3, to provide BLAST annotations against NCBI's non redundant (NR) protein database release 67 followed by mapping to gene ontology terms, and Interpro domain annotation using default parameters. Contigs were then translated using CLC Genomics Workbench 5 (CLC bio, Aarhus, Denmark) to provide a six reading frame sequence database for the proteomic characterisation of venom components. In addition, contigs annotated as toxins were also analysed manually to aid proteomic identifications, with their correct open reading frames identified via sequence alignments with known toxin sequences identified via BLAST. Trimmed raw sequencing reads have been deposited in the SRA database of NCBI (http://www.ncbi.nlm.nih.gov/sra) with the BioProject identifier PRJNA347284. Assembled contigs can be found in Supplementary File 1 and BLAST2GO annotation files are available by request from the corresponding author.

### 2.3 Phylogenetic analysis of snake venom metalloproteinases

We reconstructed the evolutionary history of the SVMP gene family using Bayesian inference. Boomslang SVMP contigs exhibiting sequence data that encoded the characteristic ‘H-box’ motif of the metalloproteinase domain were aligned with representative SVMPs isolated from other snakes that were used in previous evolutionary studies [30], [31]. We used the MUSCLE algorithm [32] in MEGA v7 [33] to align the sequences in amino acid space, and we selected the most closely related ADAM (a disintegrin and metalloproteinase) gene, ADAM28 [34] from *Homo sapiens* (GenBank: NP_055080) as our outgroup sequence. Our final dataset consisted of 55 sequences and 570 amino acid positions. We next determined the optimised model of sequence evolution chosen by the Akaike Information Criterion in MEGA v7 [33] and implemented this (WAG + G) in MrBayes v3.2.3 [35]. Bayesian inference analyses were performed using four simultaneous runs with four different chains (three hot, one cold) for 10 × 10^6^ generations and sampling every 500th cycle from the chain and using default settings in regards to priors. Tracer v1.4 [36] was used to estimate effective samples sizes for all parameters (confirmed as > 200), and to construct plots of ln(*L*) against generation to verify the point of convergence (burnin); trees generated prior to this point (before 2 × 10^6^ generations) were discarded and a consensus tree constructed from those remaining.

### 2.4 Venomic analysis: isolation and proteomic characterization of the venom proteins

0.75 mg of crude, lyophilised venom was dissolved in 200 μL of 5% acetonitrile in MilliQ® (Millipore Co.) water containing 0.1% trifluoroacetic acid (TFA), centrifuged to remove debris, and separated by reverse-phase (RP) HPLC using a Teknokroma Europa Protein 300 C18 (0.4 cm × 25 cm, 5 μm particle size, 300 Å pore size) column and an LC 1100 High Pressure Gradient System (Agilent Technologies, Santa Clara, CA, USA) equipped with DAD detector and micro-Auto-sampler [37]. The flow rate was set to 1 mL/min and the column was developed with a linear gradient of 0.1% TFA in water (solution A) and acetonitrile (solution B) using the following column elution conditions: isocratically (5% B) for 5 min, followed by 5%–25% B for 10 min, 25%–45% B for 60 min, and 45%–70% for 10 min. Protein detection was carried out at 215 nm with a reference wavelength of 400 nm. Fractions were collected manually, dried in a vacuum centrifuge (Savant), and redissolved in water, and submitted to molecular mass determination using a SYNAPT® G2 High Definition Mass Spectrometry System (Waters Corp., Milford, MA, USA), and SDS-PAGE analysis in 15% polyacrylamide gels, under reducing and non-reducing conditions. Gels were stained with Coomassie Brilliant Blue R-250 (Sigma-Aldrich, St. Louis, MO, USA).

Electrophoretic protein bands were excised from a Coomassie Brilliant Blue-stained SDS-PAGE gel and subjected to in-gel reduction (10 mM dithiothreitol) and alkylation (50 mM iodoacetamide), followed by overnight sequencing-grade trypsin digestion (66 ng/μL in 25 mM ammonium bicarbonate, 10% acetonitrile; 0.25 μg/sample) in an automated processor (ProGest Protein Digestion Workstation, Genomic Solution Ltd., Cambridgeshire, UK) following the manufacturer's instructions. Tryptic digests were dried in a SpeedVac (Savant™, Thermo Scientific Inc., West Palm Beach, FL, USA), redissolved in 15 μL of 0.1% formic acid in water, and submitted to LC-MS/MS. To this end, tryptic peptides were separated by nano-Acquity UltraPerformance LC® (UPLC®, Waters Corporation, Milford, MA, USA) using BEH130 C18 (100 μm × 100 mm, 1.7 μm particle size) column in-line with a SYNAPT® G2 High Definition Mass Spectrometry System (Waters). The flow rate was set to 0.6 μL/min and the column was developed with a linear gradient of 0.1% formic acid in water (solution A) and 0.1% formic acid in acetonitrile (solution B), isocratically 1% B for 1 min, followed by 1%–12% B for 1 min, 12%–40% B for 15 min, 40%–85% B for 2 min. Doubly- and triply-charged ions were selected for collision-induced dissociation (CID) MS/MS. Fragmentation spectra were interpreted (a) manually (*de novo* sequencing); (b) using the on-line form of the MASCOT program at http://www.matrixscience.com against NCBInr database, a comprehensive, non-identical protein database compiled from GenBank CDS translations, PIR, SwissProt, PRF, and PDB; and (c) processed in Waters Corporation's ProteinLynx Global (PLG) SERVER 2013 version 2.5.2. (with Expression version 2.0) and the generated .pkl peak list files searched against the *D. typus* transcriptomic dataset described in this paper. MS/MS mass tolerance was set to ± 0.6 Da. Carbamidomethyl cysteine and oxidation of methionine were selected as fixed and variable modifications, respectively. The cut-off for MASCOT reporting was set to the top 10 hits and all MASCOT identifications were manually verified. Amino acid sequence similarity searches were performed against the NCBInr and UniProtKB databases using the BLASTP program implemented in the WU-BLAST2 search engine at http://www.bork.embl-heidelberg.de.

The relative abundances (expressed as percentage of the total venom proteins) of the different protein families were calculated as the ratio of the sum of the areas of the reverse-phase chromatographic peaks containing proteins from the same family to the total area of venom protein peaks in the reverse-phase chromatogram [38], [39]. When more than one protein band were present in a reverse-phase HPLC fraction, their proportions were estimated by densitometry of Coomassie-stained SDS-polyacrylamide gels using ImageJ version 1.47 (Free Software Foundation, Boston, MA, USA) (http://rsbweb.nih.gov/ij). Conversely, the relative abundances of different proteins contained in the same SDS-PAGE band were estimated based on the relative ion intensities of the three most abundant peptide ions associated with each protein by MS/MS analysis. Finally, protein family abundances were estimated as the percentages of the total venom proteome.

### 2.5 Antivenomics

A second-generation antivenomics approach [40] was applied to examine the paraspecific immunoreactivity of CroFab™ (BTG International Inc., West Conshohocken, PA, USA) antivenom against *Dispholidus typus* venom. CroFab™ is a preparation of ovine Fab (monovalent) immunoglobulin fragments obtained from the blood of healthy sheep immunized with the following North American snake venoms: *Crotalus atrox* (Western diamondback rattlesnake), *C. adamanteus* (Eastern diamondback rattlesnake), *C. scutulatus* type A (Mojave rattlesnake) and *Agkistrodon piscivorus* (Cottonmouth or Water Moccasin). The final antivenom product is prepared by fractionating the immunoglobulin from the ovine serum, digesting it with papain, and isolating the venom-specific Fab fragments on ion exchange and affinity chromatography columns. To prepare the antivenom affinity column, 300 μL of (CNBr-activated Sepharose™ 4B matrix from GE Healthcare) matrix was packed in a Pierce centrifuge column and washed with 10 matrix volumes of cold 1 mM HCl followed by two matrix volumes of 0.2 M NaHCO_3_, 0.5 M NaCl, pH 8.3 (coupling buffer) to adjust the pH of the column to 7.0–8.0. Antivenom was dialysed against MilliQ® water, lyophilised, and reconstituted in coupling buffer. The concentration of the antivenom stock solution was determined spectrophotometrically using an extinction coefficient of 1.36 for a 1 mg/mL concentration of Fab at 280 nm using a 1 cm light pathlength cuvette. 5.3 mg of polyvalent antivenom were dissolved in a half matrix volume of coupling buffer and incubated with the matrix for 4 h at room temperature. Antivenom coupling yield, estimated measuring A_280_ before and after coupling of the antivenom and using the Beer-Lambert Law, was 5.1 mg. After the coupling, any remaining active groups were blocked with 300 μL of 0.1 M Tris–HCl, pH 8.5 at room temperature for 4 h. The column was alternately washed with three 300 μL volumes of 0.1 M acetate containing 0.5 M NaCl, pH 4.0–5.0, and three 300 μL volumes of 0.1 M Tris–HCl, pH 8.5; repeated 6 times. The column was then equilibrated with 5 volumes of working buffer solution (20 mM phosphate buffer, 135 mM NaCl, pH 7.4; PBS). For the immunoaffinity assay, increasing amounts (50 μg, 75 μg and 100 μg) of *D. typus* venom were dissolved in half matrix volumes of PBS and incubated with the affinity matrix for 1 h at room temperature using an orbital shaker. As a specificity control, 300 μL of CNBr-activated SepharoseTM 4B matrix was incubated with venom and the control column was developed in parallel to the immunoaffinity experiment. Non-retained fractions were collected with 5 matrix volumes of PBS, and the immunocaptured proteins were eluted with 5 matrix volumes of elution buffer (0.1 M glycine-HCl, pH 2.0) and neutralised with 150 μL 1 M Tris–HCl, pH 9.0. The non-retained and the immunocaptured venom fractions were lyophilized, reconstituted in 40 μL of MilliQ® water, and fractionated by reverse-phase HPLC using a Discovery® BIO Wide Pore C_18_ (15 cm × 2.1 mm, 3 μm particle size, 300 Å pore size) column using an Agilent LC 1100 High Pressure Gradient System equipped with a DAD detector. The column was developed at a flow rate of 0.4 mL/min and proteins eluted with a linear gradient of 0.1% TFA in MilliQ® water (solution A) and 0.1% TFA in acetonitrile (solution B): isocratic at 5% solution B for 1 min, followed by 5–25% solution B for 5 min, 25–45% solution B for 35 min, and 45–70% solution B for 5 min. Protein was detected at 215 nm with a reference wavelength of 400 nm.

## 3 Results and discussion

We first used a transcriptomic approach to characterise the toxin genes expressed in the venom gland of *D. typus*, thus representing the lethal arsenal that produced the proteins ultimately responsible for the death of Prof. K.P. Schmidt. We next applied proteomic analysis to uncover the venom's weaponry, and the integration of these two data types allowed us to correlate the symptoms experienced by the envenomed herpetologist with the composition of the lethal venom employed by *D. typus*.

### 3.1 Transcriptome profile of the D. typus venom gland

**Fig. 1:**
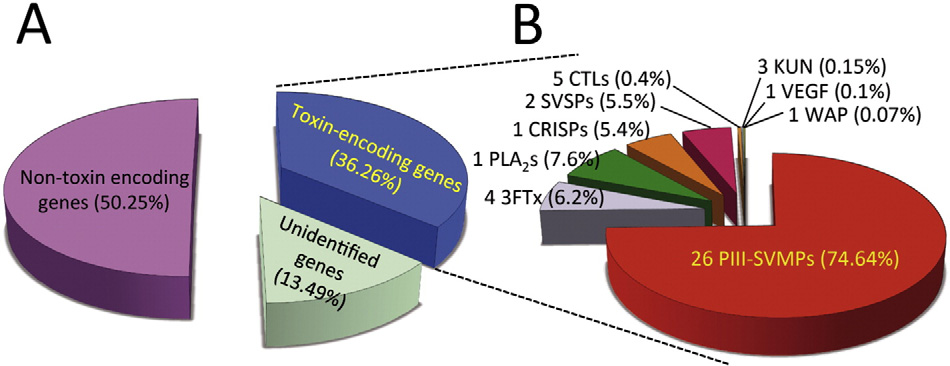
Summary statistics for the venom gland transcriptome of *D. typus*. Panel A) The relative expression of toxin-encoding genes, non-toxin encoding genes and unidentified genes detected in the venom gland. Panel B) A breakdown of the relative expression of toxin encoding genes present in the venom gland transcriptome. 3FTx, three-finger toxin; PLA_2_, phospholipase A_2_; CRISP, cysteine-rich secretory protein; SVSP, snake venom serine proteinase; CTL, C-type lectin-like; KUN, Kunitz-type inhibitor; VEGF, vascular endothelial growth factor; WAP, waprin; PIII-SVMP, snake venom metalloproteinase of class PIII.

The boomslang venom gland transcriptome resulted in the assembly of ~ 7.7 million paired-end reads into 3849 contigs with an average length of 673 bp (300–7199 bp). Annotation of these putative genes revealed 44 contigs that exhibited similarity with previously described snake venom toxin types, particularly to those reported as major or minor venom components in the venoms or the venom glands of non-front-fanged snakes from the subfamilies Colubrinae, Dipsadinae, and Natricinae [41], [42]. Combined, these transcripts accounted for 36.26% of the total gene expression identified in the venom gland (Fig. 1A). A total of 2860 contigs were found to encode for non-toxin-related genes (50.25% of expression) and 943 contigs had no annotation match (13.49%) (Fig. 1A).

**Fig. 2:**
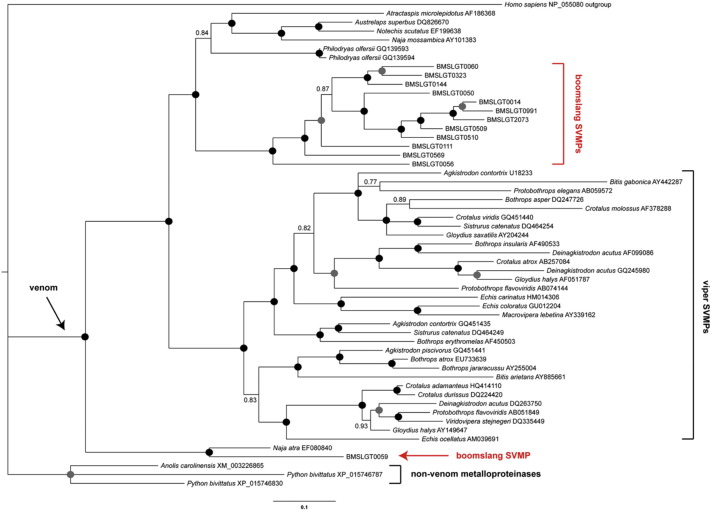
Bayesian inference phylogenetic analysis of *D. typus* snake venom metalloproteinase (SVMP) genes. *D. typus* genes are highlighted by red annotations and the origin of SVMPs indicated by the arrow labelled venom. Black circles indicate Bayesian posterior probabilities (bpp) of 1.00 and grey circles bpp of ≥ 0.95.

Of the toxin types identified in the venom gland the snake venom metalloproteinases were by far the most abundant in terms of both contig numbers (26) and expression level (74.64% of all toxin-encoding genes) (Fig. 1B). However, many of these contigs were partial in length and non-overlapping, therefore this total contig number is likely to be an overestimation of the number of SVMP genes actually expressed in the *D. typus* venom gland. All of the SVMP contigs we identified were members of the P-III class, which is unsurprising considering that P-II and P-I SVMPs have only previously been detected from viperid snakes [43], [44]. It is worth noting that we did not find any evidence of any atypical truncated SVMPs such as those previously described from the non-front-fanged snake *Psammophis mossambicus* (family Lamprophiidae) [45]. Phylogenetic analysis of boomslang SVMPs with representative orthologs from other taxa revealed that all but one of the boomslang SVMPs group together in a clade sister to that containing SVMPs from the xenodontid snake *Philodryas olfersii*, the atractaspid *Atractaspis microlepidotus* and various elapid snakes (Fig. 2). Interestingly, one of the boomslang SVMPs (contig 0059) appears to be extremely basal, and groups with an SVMP recovered from *Naja atra* at the very base of the toxin radiation (Fig. 2). This data suggests that SVMP-encoding genes may have duplicated prior to the divergence of viperid snakes from the remaining caenophidians, although the low-level expression of this gene in the boomslang, in comparison with the others, suggests that it may be of lesser importance regarding its contribution to boomslang venom toxicity.

The remaining toxin genes identified in the transcriptome were members of the 3FTx, PLA_2_, CRISP, snake venom serine protease (SVSP), C-type lectin (CTL), Kunitz-type protease inhibitor (KUN), vascular endothelial growth factor (VEGF) and waprin (WAP) toxin families (Fig. 1B). Of these, only the 3FTx, PLA_2_, CRISP and SVSP toxin families exhibited expression levels of > 1% of the toxin-encoding genes identified in the transcriptome. We found four contigs annotated as 3FTx, two of which were lowly expressed and matched 3FTx-DIS4 from *D. typus* (0.21% and 0.12%), and two of which were more abundant (4.36% and 1.52%) and showed similarity to 3FTx-DIS2 from *D. typus* and also Denmotoxin from *Boiga dendrophila* [46], [47]. We identified a single PLA_2_ contig that represented 7.56% of all toxin encoding genes, making it the third most abundant toxin contig in the venom gland transcriptome. This toxin showed similarity to the PLA_2_ IIE sub-class of phospholipases previously identified in the venom of other colubroid snakes [48] and we did not recover any other phospholipase genes that exhibited similarity to the IB or IIA classes of PLA_2_s that are canonically associated with elapid and viperid snake venoms, respectively [49]. The remaining toxin types, CRISPs and SVSPs, were found expressed at comparable levels (5.40% and 5.46%), with CRISPs encoded by a single contig and the SVSPs by two; one of which was abundant (5.27%) and the other a truncated variant with low-level expression (0.19%).

### 3.2 The venom proteome of *D. typus*

**Fig. 3:**
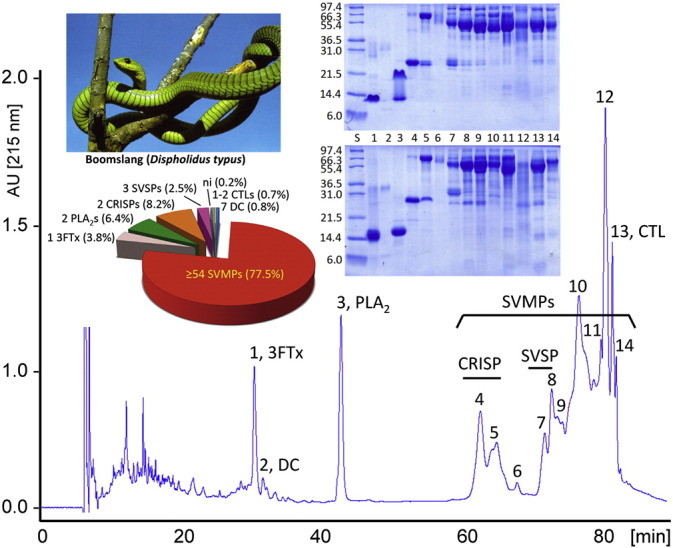
Reverse-phase HPLC separation and relative quantification of the venom proteins of *D. typus*. Fractions were collected manually and analysed by SDS-PAGE (insets) under non-reduced (upper panels) and reduced (lower panels) conditions. Protein bands were excised and characterised by LC-nESI-CID-MS/MS (Supplementary Table S1). The pie chart displays the relative abundance (in percentage of total venom proteins) of the toxin families identified in the venom. DC, disintegrin-cysteine-rich domain; ni, not identified; other acronyms as in the legend of Fig.1. Reverse-phase HPLC separation and relative quantification of the venom proteins of *D. typus*. Fractions were collected manually and analysed by SDS-PAGE (insets) under non-reduced (upper panels) and reduced (lower panels) conditions. Protein bands were excised and characterised by LC-nESI-CID-MS/MS (Supplementary Table S1). The pie chart displays the relative abundance (in percentage of total venom proteins) of the toxin families identified in the venom. DC, disintegrin-cysteine-rich domain; ni, not identified; other acronyms as in the legend of Fig.1.

The venom proteome of adult *D. typus* was characterised and quantified using reverse-phase HPLC separation (Fig. 3, Supplementary Table S1) and peptide-centric tandem mass spectrometry-based bottom-up venomics [37], [38]. Nano-electrospray ionisation mass spectrometry (nESI-MS/MS) identified proteins belonging to 7 different snake venom protein families (Fig. 3, Supplementary Table S1), including a 3FTx (Dis-2 [ABU68481] [47], residues 22–94; M_ave_: 8437.7 Da); two type IIE D-49 PLA_2_ molecules (AFH66958, residues 24–147, M_ave_: 13,642.1 Da; and an isoform of AFH66959, residues 24–147, M_ave_: 13,665.1 Da) [48]; two isoforms of CRISP molecules (M_ave_: 24,987.5 Da; residues 17–237 of Q2XXQ4 and Q2XXQ5 [50], with one M_ox_); three SVSPs; one or two CTLs; a variety of SVMPs; and seven Disintegrin-like/Cysteine-rich (DC) proteolytic fragments of PIII-SVMPs. Reverse-phase chromatography, SDS-PAGE and MS/MS-derived tryptic peptide approaches provided evidence for the translation of 19 SVMP-encoding transcripts, demonstrating that our transcriptome assembly may have only resulted in a modest overestimation of SVMP genes. *D. typus* SVMPs eluted in RP-HPLC fractions 4–14 and have molecular masses in the range of 21–97 kDa. On the other hand, the fact that the same SVMP eluted in different chromatographic fractions and display different molecular mass by SDS-PAGE analysis indicated the existence of different proteoforms (“different molecular forms in which the protein product of a single gene can be found”; e.g. glycoforms) or closely related toxin isoforms (“forms of protein molecules arising from the same gene”; i.e., full-length PIII-SVMPs and proteolytically processed SVMPs that have released their C-terminal DC domains) [51], [52]. In this regard, five out of the seven DC domains identified in RP-HPLC fractions 1 and 2 (Fig. 3, Supplementary Table S1) also formed part of full-length SVMPs, while the other two were only found as processed DC domains and no evidence for the metalloproteinase part of the protein was gathered. The proteomic data suggest the existence of 54 proteo/iso-SVMPs, 40 protein species of molecular masses 97–40 kDa and 14 SVMPs of molecular masses between 33 and 21 kDa. Collectively, these proteins account for 77.5% of the *D. typus* venom proteome, with the majority (53% of the venom proteome) corresponding to the 52–66 kDa SVMPs (Fig. 3, Supplementary Table S1).

With the exception of a lack of evidence for the presence of KUN, VEGF and WAP transcripts in the boomslang venom proteome, the proteomic- (Fig. 3) and transcriptomic-derived (Fig. 1) toxin compositions agree remarkably well.

### 3.3 Boomslang venom composition-activity correlations

The boomslang that fatally bit K.P. Schmidt was a young specimen, and only one fang penetrated the skin to a depth of 3 mm. Unalarmed by the bite, he decided to document the nature of his health in response to a potential envenoming. K.P. Schmidt's meticulous “death notes”, published by C.H. Pope in 1958 [1], represents a very unique and personal look at the effects of a boomslang bite while being objective about it. Schmidt's diary stopped the following morning when he believed he was recovering. However, after noon he had difficulty breathing and died soon after, shortly before 3:00 pm, due to respiratory paralysis. The autopsy revealed that his difficulty in breathing was due to bleeding into the lungs. The examination also showed hemorrhaging in the renal pelvis and the small intestine which accounts for Schmidt's documentation of blood in his urine and bowels. We now know that several colubroid taxa can cause lethal or life threatening envenoming in humans (see Table 4 in [53]), and the coagulopathic and haemorrhagic character of boomslang venom has been demonstrated in experimental animals [54]. Our present comprehensive report on the venom proteome of the species that killed Schmidt gives us the opportunity of trying to correlate venom composition with the causes of his fatal outcome. However, caution should be taken when assigning bioactivities to colubroid toxins based on homology with toxins from snakes of other families, as the biochemistry of colubroid toxins may be very different from those of the well-studied front-fanged snakes. Many non-front-fanged snake venoms exhibit modal or low lethal potencies in the murine model but instead have high toxicity and potency in some avian and lizard models [55], [56]. Thus, members of the Elapidae 3FTx family exhibit a wide variety of pharmacological effects in laboratory animals (e.g. mice), including postsynaptic neurotoxicity, cytotoxicity, cardiotoxicity, and anticoagulant, and antiplatelet activities [57], [58]. α-Colubritoxin, isolated from the Asian ratsnake *Coelognathus radiatus*, represented the first 3FTx reported from a non-front-fanged snake venom [59]. This potent postsynaptic neurotoxin exhibits structural and functional homology to elapid nicotinic acetylcholine receptor antagonistic 3FTXs [59]. However, other 3FTxs functionally characterised from members of the subfamily Colubrinae, such as denmotoxin (*Boiga dendrophila*) and irditoxin (*B. irregularis*), exhibit taxon-specific activities [46], [60]. The weak (< 50%) amino acid sequence identity of the *D. typus* 3FTx with elapid homologs, and the fact that despite being an abundant venom toxin, the bite did not produce apparent signs of neurotoxicity, suggests that this boomslang 3FTx may represent another taxon-specific toxin. By the same token, although their high structural conservation and broad distribution among many front and non-front-fanged snakes suggests a significant biological role in venom [12], [41], [61], the relevance of non-front-fanged snake venom CRISPs in human envenomings is not yet clear. Patagonin, a CRISP isolated from the venom of *P. patagoniensis*, showed necrotic activity toward murine gastrocnemius muscle when injected intramuscularly at doses of 43 and 87 μg [62], possibly by binding to ion channels [63]. However, at 20 μg, patagonin did not induce oedema or hemorrhage, and it had no effect on the aggregation of human platelets or platelet-rich plasma (at concentrations as high as 100 nM).

A number of snake venom toxins interact with components of the human haemostatic system affecting the blood coagulation cascade and platelet aggregation, including disintegrins, PLA_2_s, CTL-like molecules, SVSPs, and SVMPs [64], [65], [66]. Except disintegrins, which are proteolytically derived from PII-SVMPs and therefore only expressed in venoms of Viperidae [67], [68], the other toxin types targeting the haemostatic system comprise > 87% of the boomslang venom proteome (Fig. 3), which correlates well with the haemorrhagic pathology observed in Schmidt's autopsy.

High levels of PLA_2_ activity have previously been reported in *D. typus* venom [69]. The venoms of many terrestrial and marine Australo-Papuan elapid snakes are also rich in D49-PLA_2_ toxins and cause a range of actions including presynaptic neurotoxicity, myotoxicity, anticoagulant, anti-platelet, hypotensive, haemorrhagic and myonecrotic activities [70], [71]. Relevant to this discussion is the recent report that *P. papuanus* venom induced lethality, intravascular hemolysis, pulmonary congestion and edema, and anticoagulation after intravenous injection in mice, and these effects were mainly due to the action of PLA_2_s [72].

The CTLs identified in the boomslang venom show 60% sequence identity to a vast number of snake venom CTLs, particularly contigs T1304, T3784 and T1088 which exhibit 87%, 84% and 94% amino acid sequence identity to *B. irregularis* CTL-6 [JAS04587] and CTL-5 [JAS04588] [73], and lectoxin-Thr1 [A7X3Z0] from *T. jacksonii* [47] respectively. CTLs inhibit or activate platelets by binding to various receptors [74]. Activation represents an efficient way to reduce platelet function because activated platelets are removed from the circulation producing thrombocytopenia. The mechanisms by which *D. typus* CTLs affect hemostasis has not yet been studied.

Serine proteases (SVSPs), include a variety of venom enzymes which interfere with vertebrate hemostasis and have previously been documented from colubroid venoms. In addition to *D. typus*, bites from several colubroids have resulted in prolonged clotting times (*Rhabdophis tigrinus*), prolonged defibrination (*R. subminiatus*), prothrombin activation (*Thelotornis capensis*; *R. tigrinus*; *R. subminiatus*) and other disturbances of hemostasis [12]. Bites from these species cause consumptive coagulopathy and haemorrhagic diathesis, complicated in some cases by acute kidney injury, and are designated “Hazard Level 1” [53]. Fibrin(ogen)olytic serine proteases have isolated from the venom of *Philodryas olfersii* [75]. *D. typus* venom serine proteinases exhibit 75–78% amino acid sequence identity with a number of proteolytic enzymes from colubroid (*P. olfersii* Q09GK1 [76]) and elapid venoms, including the fibrin(ogen)olytic enzymes Q5MCS0 (*Hydrophis hardwickii*) [77] and A8QL56 (*Ophiophagus hannah*) [78].

For most colubroid species, especially in the subfamily Dipsadinae, snake venom metalloproteinases (SVMPs) are dominant components in transcriptomes and proteomes [41]. Our results here demonstrate that the vast majority of toxin genes expressed in the venom gland transcriptome (~ 75%) and toxin proteins detected in secreted venom (~ 78%) are SVMPs. All sequences described in non-front-fanged snakes to date belong to (or are derived from) the P-III class of SVMPs [79] and we find the same with the boomslang. *D. typus* PIII-SVMPs show 66–69% identity to *Boiga irregularis*, *Hypsiglena* sp. JMG-2014, *Phalotris mertensi* and *Philodryas olfersii* SVMPs. They also have significant homology (50–65%) to elapid and viperid PIII-SVMP, including 61–63% identity to Factor X activators from *Daboia russelii russelii* (ADJ67475) and *Macrovipera lebetina* (Q7T046).

A 67 kDa prothrombin activator (“coagulant principle”, “procoagulant”) from *D. typus* venom has been partially characterised [80]. This protein is likely the SVMP termed dispholysin A, which was previously reported to cross-react with polyclonal antibodies to the *Bothrops jararaca* venom metalloprotease jararhagin [22]. A central feature of the clinical pathology produced by envenomations by procoagulant SVMPs is a DIC-like (disseminated intravascular coagulation-like) syndrome [65]. This clinical pathology is characterised by depletion of fibrinogen from the blood as a result of prothrombin activation, resulting in a net effect of incoagulable blood. About 50% of all *D. typus* SVMPs have the same molecular mass as dispholysin A, and it is therefore tempting to speculate that this procoagulant SVMP may have been at least partially responsible for potentiating the bleeding pathology observed in the case of K.P. Schmidt. Boomslang venom also contains a number of other PIII-SVMPs, and it seems reasonable to state that it is likely some of these are haemorrhagic, considering many PIII-SVMPs are potently haemorrhagic [81], [82]. The combined consumption and subsequent exhaustion of coagulation proteins and platelets (from ongoing activation of coagulation) and the action of haemorrhagic SVMPs may have resulted in widespread clotting and bleeding. Unveiling the functional features of the full complement of boomslang SVMPs deserves a detailed toxicovenomics investigation [83], [84].

### 3.4 Antivenomic assessment of the immunoreactivity of CroFab™ towards boomslang venom proteins

**Fig. 4:**
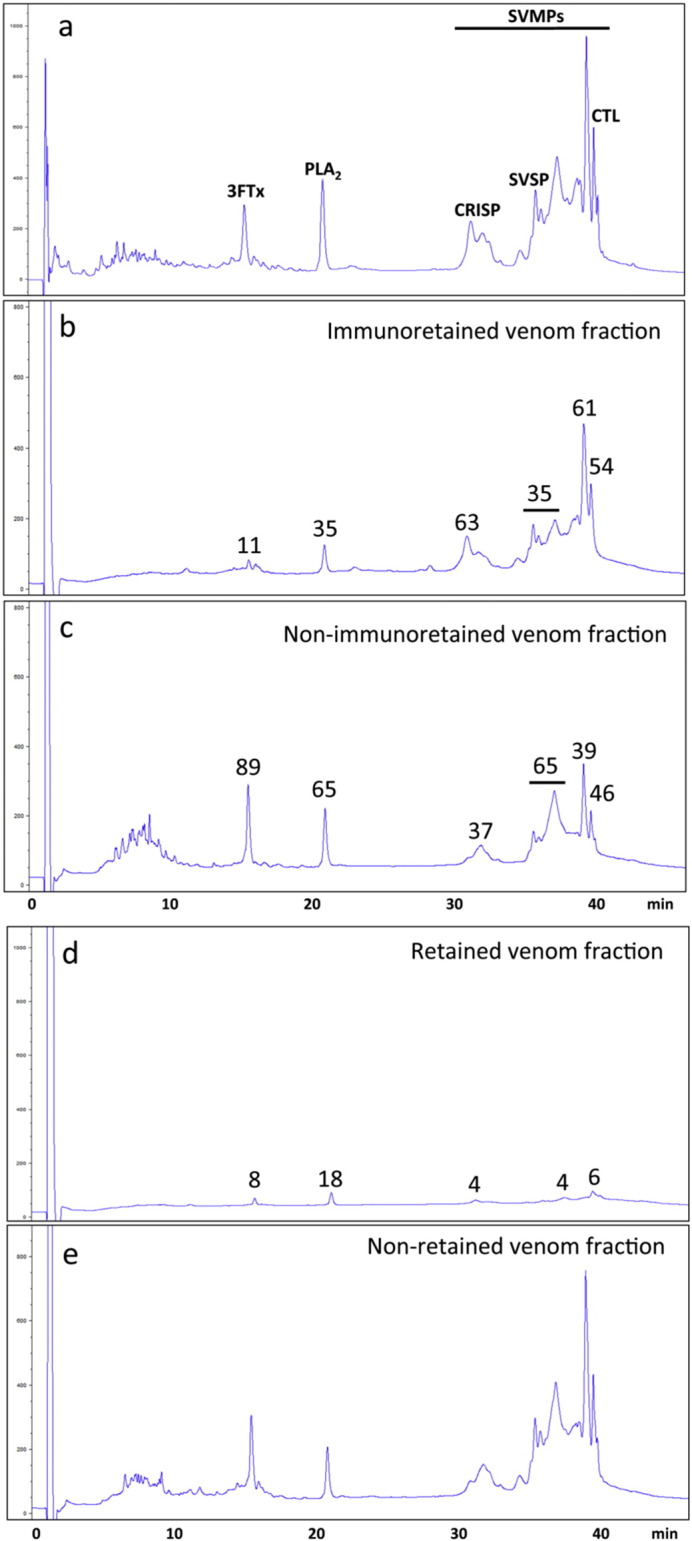
Immunoaffinity antivenomics analysis of *D. typus* venom against CroFab™ antivenom. Panel a, reference RP-HPLC separation of proteins of *D. typus* venom. Protein classes identified in the different chromatographic fractions are highlighted. Panels b and c display, respectively, reverse-phase separations of the immunocaptured and the non-bound column fractions recovered after incubating 100 μg of venom with 300 μL of Sepharose-immobilised (5.1 mg) CroFab™ antivenom. Panels d and e show, respectively, reverse-phase HPLC separations of the venom components recovered, respectively, in the bound and non-bound fractions of a mock Sepharose 4 Fast Flow matrix column (matrix control). Column eluates were monitored at 215 nm and quantified by comparing the areas of homologous peaks in the two fractions. Numbers indicate the percentage of venom component in the chromatographic fraction.

We tested the ability of a commercially available N. American antivenom (CroFab™) to immunocapture boomslang venom proteins using an antivenomics approach. Whilst this antivenom was not available at the time of K.P. Schmidt's death, having since superseded the historical Wyeth-Ayerst Laboratories Antivenin (Crotalidae) Polyvalent (ACP) that was available at that time, a number of the venoms used for producing the two antivenoms are shared (e.g. *C. adamanteus*, *C. atrox*). Perhaps surprisingly, when considering the degree of taxonomic separation between colubroid (i.e. *D. typus*) and viperid snakes (i.e. *C. adamanteus* and *C. atrox*), CroFab™ showed cross-immunoreactivity towards most boomslang venom proteins, albeit with varying degrees of affinity (Fig. 4). CRISP and the major SVMP peaks (11–14, Fig. 3) were efficiently (51–64%) immunoretained, while the PLA_2_ molecule, SVSPs, and SVMPs eluting in peak 11 were immunoretained to a lesser (35%) extent (compare panels b and c of Fig. 4). On the other hand, the small amounts (8%) of immunoretained 3FTx (Fig. 4, panel b) were not significantly different from those of the same chromatographic fractions non-specifically retained in the control column (mock chromatographic matrix) (Fig. 4, panel d). Although a correlation between the level of immune recognition gathered from antivenomics with the *in vivo* pre-clincial neutralization capacity of an antivenom is not straightforward, since both experiments involve radically different protocols, our previous experience shows that even a moderate immunocapturing capability of ~ 20%–25% can correlate with a good outcome in pre-clinical *in vivo* neutralization tests [83].

## 4 Concluding remarks

Prof. K.P. Schmidt succumbed to the bite of a snake considered by the herpetologists of the time as harmless to humans. Since then, only a handful of studies on the biological activities of the venom of this colubroid have been reported [3], [14], [15], [16], [21], [22], and only a few cases of human and veterinary envenomings by *D. typus* have been documented [8], [9], [15], [16], [17], [84], [85], [86], [87], [88], [89], [90], [91]. The present work is the first to address a comprehensive proteomic characterization of *D. typus* venom, allowing us to correlate its toxin composition with the toxic activities of this venom. At the time the unfortunate accident occurred no specific antivenom against boomslang venom was available. However, a whole IgG antivenom, Antivenin (Crotalidae) Polyvalent (ACP), was introduced in USA by Wyeth-Ayerst Laboratories in 1954 [92]. The ACP antivenom was generated in horses against a mixture of venoms from the Eastern diamondback (*C. adamanteus*), Western diamondback (*C. atrox*) and South American (*C. durissus terrificus*) rattlesnakes, and the fer-de-lance (*Bothrops asper*), and has been an important part of snakebite therapy for 35 years, particularly for the life-threatening or coagulopathic manifestations of crotaline snakebite in USA [93]. As mentioned earlier, the Wyeth-Ayerst antivenom has since been replaced by CroFab™ [Crotalidae Polyvalent Immune Fab (ovine)], a preparation of ovine Fab (monovalent) immunoglobulin fragments obtained from the blood of healthy sheep immunized with one of the following North American snake venoms: *C. atrox*, *C. adamanteus*, *C. scutulatus* type A and *A. piscivorus*. Venoms of *C. adamanteus*, *C. atrox*, *B. asper*, and *A. piscivorus* are characterised by a high content of haemorrhagic SVMPs [94], [95], [96]. Full-length amino acid sequences of boomslang venom gland SVMP-encoding transcripts exhibit ~ 61% identity with a number of PIII-SVMPs from venoms of *Agkistrodon* and *Crotalus* species, suggesting that the polyvalent ACP antivenom could have exhibited at least some immunoreactivity against the boomslang SVMPs. This assumption is supported by the well documented immunoreactivity among SVMPs from phylogenetically distant snakes [97], [98], including the previously reported cross-reaction of *D. typus* SVMP dispholysin A with polyclonal antibodies generated against the SVMP jararhagin from *B. jararaca* [22]. Moreover, the efficient immunodepletion of some boomslang SVMPs in our CroFab™ affinity column (Fig. 4) further underpins this hypothesis.

So, what killed KP Schmidt? The boomslang is a timid snake, and bites generally occur only when people attempt to handle, catch or kill the animal, and thus K.P. Schmidt's fatal outcome was presumably due to an accumulation of unfortunate circumstances. On the one hand, the carefree handling of the snake (“*I took it from Dr. Robert Inger without thinking of any precaution, and it promptly bit me on the fleshy lateral aspect of the first joint of the left thumb. The mouth was widely opened and the bite was made with the rear fangs only, only the right fang entering to its full length of about 3 mm*”), on the other the false prevailing belief in the scientific, medical and popular literature of that time that considered colubroids as harmless snakes and consequently that bites by rear-fanged snakes did not pose medical risks to humans, may have contributed to K.P. Schmidt refusing to receive medical attention. And, if he had sought medical treatment, would he had been treated with the available anti-Crotalidae polyvalent antivenom? There was no clinical studies that hinted to the possible effectiveness of ACP antivenom in a boomslang envenoming, and even to this day, we are unaware of any reports of viper antivenoms being demonstrated to be pre-clinically or clinically efficacious for treating systemic envenoming caused by colubroid snakes.

Advancements in venomic analysis have resulted in the ability to generate comprehensive profiles of a large number of snake venoms [99], including a growing number of rear-fanged snakes [41], [42]. Biochemical and pharmacological studies have also deepened our knowledge about rear-fanged snake venoms, and revealed that a number of species (*D. typus*, *Thelotornis capensis*, *Rhabdophis tigrinus*, *R. subminiatus*, *Balanophis ceylonensis*, *Philodryas olfersii*, and *Tachymenis peruviana*) are able to deliver lethal quantities of venoms and cause human fatalities [10], [12], [53], [100], [101], [102], [103], [104], [105], [106], [107]. Increasing awareness of life-threatening envenomings from rear-fanged snake bites should lead to an increased interest in research focused on these venoms, which in turn should contribute to prevent fatal human accidents, such as that unfortunately suffered by Karl Patterson Schmidt.

The following is the supplementary data related to this article.

Supplementary Table S1 MS/MS identification and quantification of the boomslang (Dispholidus typus) venom proteins separated by RP-HPLC and SDS-PAGE as in Fig. 2. Supplementary Table S1

## Transparency document

Transparency document. Image 1
